# Unraveling the Potential of Orange Pulp for Improving Laying Rate, Egg Quality, Oxidative Stability, Fatty Acids Composition, and Reproductive Tract Morphology of Laying Hens

**DOI:** 10.3390/ani13132199

**Published:** 2023-07-05

**Authors:** Eman Hussein, Rashed A. Alhotan, Alia Ebrahim, Shaimaa Selim

**Affiliations:** 1 Department of Poultry and Fish Production, Faculty of Agriculture, University of Menoufia, Shibin El-Kom 32514, Egypt; 2 Department of Animal Production, College of Food and Agriculture Sciences, King Saud University, Riyadh 11451, Saudi Arabia; 3 Jiangsu Key Laboratory for Microbes and Genomics, School of Life Sciences, Nanjing Normal University, Nanjing 210023, China; 4 Department of Nutrition and Clinical Nutrition, Faculty of Veterinary Medicine, Menoufia University, Shibin El-Kom 32514, Egypt

**Keywords:** dried orange pulp, laying hens, egg quality, serum metabolites, reproduction, oxidative stability, egg shelf life

## Abstract

**Simple Summary:**

Orange pulp is an industrial byproduct that contains naturally active components, such as phenolic acids and flavonoids. Therefore, the objectives of this trial were to demonstrate the effects of dietary dried orange pulp (DOP) on the laying performance, egg quality, oxidative stability, yolk fatty acid profile, serum biochemistry, and reproductive tract morphology of laying hens. The body weight gain, feed intake, egg production, egg weight, egg mass, and feed conversion ratio of laying hens fed the DOP diets were greater than those fed the control diet. Eggs obtained from the DOP groups had a heavier shell weight, shell thickness, and greater yolk color score. Dietary DOP enhanced the egg yolk proportions of beneficial fatty acids, whereas the contents of yolk saturated fatty acids, cholesterol, and triglycerides were decreased. After storage for 40 days, the eggs obtained from the DOP hens had a better antioxidant capacity and lower lipid peroxidation rate. The ovary, oviduct, uterus, and follicle weights of hens receiving diets containing DOP were heavier than those of the control hens. In conclusion, dietary DOP up to 100 g/kg of feed improves the laying performance, health status, antioxidant capacity, egg nutritive value, and egg shelf life in laying hens.

**Abstract:**

The current study aimed to demonstrate the effects of dietary dried orange pulp (DOP) on the laying performance, egg quality, antioxidant status, yolk fatty acid composition, serum biochemistry, and reproductive tract morphology of laying hens. A total of 200 Lohman Brown Lite laying hens were randomly allotted into 4 dietary treatments with 10 replicates each. The experimental treatment groups were the control group, a basal diet containing 50 g DOP/kg feed (DOP_5%_), a basal diet containing 70 g DOP/kg feed (DOP_7%_), and a basal diet containing 100 g DOP/kg feed (DOP_10%_). Data were statistically analyzed by one-way ANOVA following a completely randomized design, and the incremental levels of dietary DOP were tested by orthogonal polynomial contrasts. The body weight gain, feed intake, egg production%, egg weight, egg mass, and feed conversion ratio of laying hens fed the DOP_7%_ and DOP_10%_ diets were greater (*p* < 0.01) than those fed the control diet. Eggs obtained from the DOP_7%_ and DOP_10%_ groups had a heavier shell weight% and shell thickness, as well as a greater yolk color score (*p* < 0.01; linear, *p* < 0.01). Dietary DOP improved the egg yolk concentrations of PUFA, n-3 PUFA, and n-6 PUFA (linear, *p* < 0.001; quadratic, *p* < 0.05), whereas the content of SFA was reduced (*p* < 0.001; linear, *p* < 0.001). The egg yolk cholesterol and triglyceride levels were linearly decreased (*p* < 0.001) with the inclusion of DOP in the diets of hens. After storage for 40 days, the malondialdehyde (MDA) contents in the egg yolk were reduced, whereas the glutathione peroxidase content was increased (*p* < 0.01) due to dietary DOP. The DOP_7%_ and DOP_10%_ hens had an obvious reduction in the levels of serum total lipids, total cholesterol, triglycerides, low-density lipoprotein, and MDA, whereas high-density lipoprotein and GPx levels were increased (*p* < 0.01) compared with those fed the control diet. The relative weights of the ovary, oviduct, uterus, and follicle of hens receiving diets containing 7% and 10% DOP were heavier (*p* < 0.01) than those of the control hens. Moreover, the number of large yellow follicles was increased (*p* < 0.001; linear, *p* < 0.001) in the hens-fed diets containing 7% and 10% DOP. In conclusion, dietary DOP at up to 100 g/kg of feed improves laying performance, health status, antioxidant capacity, egg nutritive value, and egg shelf life in laying hens.

## 1. Introduction

Over the last decade, a continuous increase in poultry production costs has been recognized due to the elevated prices of feed ingredients. Accordingly, alternative low-input feeding approaches based on agro-industrial byproducts are fundamental to lessening poultry nutrition expenditures [1,2,3]. At the same time, animal products are currently expected to meet consumers’ nutritional needs and protect them from metabolic health problems. Nowadays, the improvement of diet composition becomes a key factor to improve the health status and welfare of animals [4], as well as enhancing productivity and performance in livestock [5,6]. Antioxidants and antimicrobial food additives from a synthetic origin are commonly used in the food industry to postpone the natural degradation and peroxidation of foods. The consumption of these additives has been linked to the incidence of carcinogenesis, and their influence on human health due to long-term consumption is still unknown [3]. Thus, seeking natural alternatives to these components is essential without inducing any detrimental health influences. Agro-industrial byproducts are a cheap source of antioxidant components, and the consumption of animal products, for instance, meat and eggs, rich in these beneficial nutraceuticals can strengthen humans’ health and immunity [2,3,7]. Moreover, the application of agro-industrial byproducts in poultry nutrition could represent a prospect to reduce environmental pollution and permit the sustainability of their beneficial ingredients in the food chain [8]. The use of agro-industrial byproducts may efficiently lessen the cost of waste processing and management [7], which is considered an interesting area in the livestock industry. 

Oranges are well-known fruits, belonging to the genus *Citrus*, with a production of more than 76 million tons worldwide in 2020, and the annual orange production in Egypt increased from 3.01 Mt in 2019 to 3.16 Mt in 2020 (FAOSTAT, 2022; https://www.fao.org/faostat/en/#data, accessed on 10 February 2023). Dried orange pulp (DOP) is an industrial byproduct produced after the juice extraction from orange fruits and drying of the remains. Orange pulp (OP) is a mixture of peel, seeds, and pulp that contains naturally active components, such as phenolic acids and flavonoids [9,10,11]. Moreover, orange peel is a good source of dietary fiber, pectin, phenolic acids, and flavonoids [10,11]. Dietary fiber is a vital source to avoid the incidence of cardiovascular diseases, diabetes, cancer, and gastrointestinal disorders [12]. Flavonoids and vitamin C present in DOP were reported to have beneficial antioxidant properties [13,14], antimicrobial actions, immune-stimulating activities [15,16,17], and chelating actions [13,14]. 

Currently, DOP is considered a cheap feedstuff in the diets of dairy cattle and fattening lambs [18], and it was reported to enhance growth performance and lessen production costs, whereas its application in poultry diets is limited [16,19,20,21]. Citrus pulp and its flavonoids have previously been applied in diets for broiler chickens, with different findings concerning the growth performance, but with positive impacts on the antioxidant properties of the meat. Dietary inclusion of dried citrus pulp (DCP) at up to 3% had a non-influence on the performance of broiler chickens [16,19]. It was shown that DCP [19] or *Citrus sinensis* peel extract [16] can decrease the abdominal fat percentage and augment gut microbiota in broiler chickens [20]. However, with higher inclusion levels, Mourao et al. [21] showed that dietary incorporation of DCP at a level of 5% to 10% decreased body gain, while increasing feed intake and reducing the feed conversion ratio (FCR) in broiler chickens. Recently, Zoidis et al. [22] observed that dietary supplementation DOP at 50 g/kg along with organic Se improved the meat oxidative stability and nutritional value without inducing any detrimental effects on the performance of broiler chickens. Nazok et al. [23] reported that the inclusion of DCP up to 12% in the diets of laying hens had no negative influence on the performance and egg quality of laying hens. Moreover, Goliomytis et al. [24] showed that dietary incorporation of DOP at 90 g per kg of feed improved the oxidative stability of eggs in laying hens, while its supplementation was associated with adverse effects on the performance and egg quality of laying hens. 

To our knowledge, very limited research is published on the effect of DOP on laying performance and egg yolk oxidative stability, and no data exist regarding the effects of graded dietary inclusion of DOP on egg quality traits, egg shelf life, yolk fatty acid (FA) composition, or reproduction morphology in laying hens. Therefore, the current study aimed to demonstrate the effects of dietary inclusion of DOP on the laying performance, egg quality, antioxidant capacity, egg shelf life, yolk fatty acid composition, and reproductive structure of laying hens. We hypothesized that the dietary inclusion of DOP could be effective in enhancing egg quality traits and antioxidant capacity without adversely impacting the performance of laying hens.

## 2. Materials and Methods

The current trial was performed under a research protocol approved by the Animal Ethics Committee, Faculty of Agriculture, University of Menoufia, Egypt, and compliant with the Egyptian Guidelines for Animal Welfare. The ethical approval number is MOAGR, 11/22.

### 2.1. Dried Orange Pulp Analysis

Dried orange pulp was obtained from Paste and Juice Company (P & J), Sadat City, Egypt. Dry matter (DM), crude protein (CP), crude fiber (CF), neutral detergent fiber (NDF), acid detergent fiber (ADF), ether extract (EE), and ash contents of DOP were determined according to the procedures of AOAC [25]. A total of 2 g of the DOP samples were extracted in 80% aqueous methanol at 25 °C on an orbital shaker (200 rpm) for 4 h. The supernatants were centrifuged, filtered, and decanted. The total phenolic content of the methanolic extract solution was measured colorimetrically using Folin–Ciocalteu reagent following Al-Farsi et al. [26], and it was presented as the equivalent mg of gallic acid/g dry weight of DOP (mg GAE/100 g DW). Methanolic extracts were used for the determination of total flavonoids. Total flavonoid content was estimated by a colorimetric assay, as described by Kim et al. [27], and it was recorded as the equivalent mg of catechin/100 g dry weight of DOP (mg CE/g DW). All chemicals were obtained from Sigma-Aldrich Co. (St. Louis, MO, USA). The nutrient composition of DOP is presented in Table 1. 

### 2.2. Experimental Design and Management

A total of 200 Lohman Brown Lite laying hens during the early phase of production (25 weeks old), with a body weight of 1310 ± 3.56 g, were randomly allotted into 4 dietary treatments with 10 replicates each (5 hens/replicate, 50 hens per group) in a completely randomized design for a period of 8 weeks (from 25 weeks old to 33 weeks old). Laying hens were equally distributed in the treatment groups based on their age (25 weeks old) and body weights (1310 ± 3.56 g, mean ± SEM). Laying hens were kept in cages of 0.44 m high × 0.30 m wide × 0.45 m deep, provided with nipple drinkers and trough feeders. Laying hens were vaccinated and managed following the breeder standards (Lohmann Brown Lite layers, Commercial Management Guide, Lohmann Tierzucht, Cuxhaven, Germany). The light program was 16 h light and 8 h darkness per day, and the temperature and relative humidity in the laying hens’ house were 25 ± 2 °C and 60–65%, respectively, during the trial period. Feed and water were supplied ad libitum during the experiment. Laying hens were checked daily for health status. The experimental treatment groups were the control group (basal diet, no DOP; control), a basal diet containing 5% DOP (DOP_5%_; 50 g DOP/kg of feed), a basal diet containing 7% DOP (DOP_7%_; 70 g DOP/kg of feed), and a basal diet containing 10% DOP (DOP_10%_; 100 g DOP/kg of feed). The experimental diets were formulated to be isocaloric and isonitrogenous and to meet the breeder’s recommendation (https://lohmann-breeders.com/files/downads/MG/Cage/LB_MG_Cage_LB-Lite_EN.pdf, accessed on 5 January 2023). The feed ingredients, proximate composition, and FA profile of the experimental diets are presented in Table 2.

### 2.3. Laying Hens Performance and Egg Quality Traits

The body weights of laying hens were determined at the beginning (25 weeks of age) and at the end of the trial (33 weeks of age). Feed intake (FI) was recorded weekly per replicate and then calculated as daily FI per bird. The number of eggs and the egg weights were recorded weekly during the trial period. Egg mass was determined by multiplying egg weight by egg production%, and the FCR was recorded as a gram of FI divided by a gram of egg mass. Mortality was recorded during the experiment. Egg quality traits, involving the albumen and yolk weights, shell thickness, and yolk color score, were measured separately in 30 fresh eggs collected randomly from each replicate during the last 3 days of week 33 of laying hens’ age. Shell thickness was measured by micrometer after the removal of shell membranes, and the mean value of three locations on the egg (at the 2 pole ends and at the middle section of the egg) was recorded. Albumen and yolk heights were performed using a standard tripod micrometer, and the caliper was used to determine yolk diameter. The egg shape index (%), egg yolk index (%), and egg yolk albumen index (%) were determined by measuring egg width and length with a caliper. The egg yolk color was determined by the Roche yolk color fan. Haugh units were calculated using the formula described in Selim and Hussein [2].

### 2.4. Reproductive Tract Morphology

At the end of the trial period (33 weeks of age), 2 laying hens, within the average BW, from each replicate were chosen and slaughtered. Directly after slaughtering, the oviduct, ovary, and associated follicular hierarchy were removed and weighted by a sensitive caliper. The number of large yellow follicles with a diameter greater than 10 mm, small yellow follicles with a diameter of 5 to 10 mm, and large white follicles with a diameter of 3 to 5 mm were recorded. The weights of the ovary, oviduct, uterus, follicle, and ovarian stroma were recorded, and their relative weights to the live body weight of laying hens were determined.

### 2.5. Serum Biochemistry

At the end of the trial (33 weeks of age), blood samples were collected in tubes without anticoagulant from the wing vein of 2 laying hens per replicate (a total of 20 blood samples per group) for serum analysis of biochemical constituents. Serum was obtained by centrifugation at 1500× *g* for 5 min, transferred into Eppendorf tubes, and stored at −20 °C until subsequent analysis. The serum samples were analyzed spectrophotometrically (spectrophotometer UV4802, Unico Co., Dayton, OH, USA) using available kits (Biosystem S.A, Costa Brava, 30, Barcelona, Spain) for determining serum total protein (https://www.clinicord.com/wp-content/uploads/pdfs/11500c.pdf, accessed on 4 September 2022), albumin, total lipids, cholesterol, triglycerides, alanine aminotransferase (ALT), and aspartate aminotransferase (AST) concentrations. The determination of blood MDA was conducted with a spectrophotometer according to the method of Esterbauer and Zollner [28] using a commercial kit (Sigma-Aldrich, St. Louis, MO, USA). The blood GPx level was also measured using commercial kits (Biodiagnostic, Giza, Egypt), according to the manufacturer’s procedures and as described by Plaser et al. [29].

### 2.6. Egg Yolk Oxidative Stability and Fatty Acid Composition

A total of 30 eggs from each treatment group at the end of the trial (33 weeks of age) were randomly selected for determining egg yolk cholesterol and triglycerides using available kits and an ultraviolet spectrophotometer UV4802 (Unico Co., Dayton, OH, USA), as described by Hammad et al. [30] and Kaya et al. [31]. For oxidative stability assessment, another set of 30 eggs from each group (3 eggs/replicate) was preserved in the refrigerator at 4 °C for 20 and 40 days. Lipid peroxidation in fresh and stored eggs was measured as thiobarbituric acid reactive substances (TBARSs) of the egg yolk, according to the method described by Botsoglou et al. [32] and Galobart et al. [33] using commercial kits (Sigma-Aldrich, St. Louis, MO, USA). Malondialdehyde (MDA) was analyzed by a spectrophotometric method, and the TBARS concentration was expressed as mg of MDA per g of egg yolk (mg/g). Glutathione peroxidase (GPx) activity was determined according to Paglia and Valentine [34] using commercial kits (Sigma-Aldrich, St. Louis, MO, USA), according to the manufacturer’s instructions. Glutathione peroxidase was expressed as mg per g of egg yolk. 

At the end of the experiment (33 weeks of age), 20 samples from each group were gathered to determine the fatty acids (FAs) profile in the egg yolk. The analysis of the egg yolk FAs was conducted by the change of oil to FA methyl esters, according to the protocol defined by Yang et al. [35] using a gas chromatograph (Model GC-14A, Shimadzu Corporation, Kyoto, Japan) with a flame ionization detector and a polar capillary column (BPX70, 0.25; SGE Incorporated, Austin, TX, USA). The FA methylated esters were established by matching their retention times with those of their standards (Sigma-Aldrich, St. Louis, MO, USA). The FAs relative percent was quantified as a percentage of the total analyzed FAs in the sample. 

### 2.7. Statistical Analysis

The Kolmogorov–Smirnov test and Levene’s test were used to determine the normality of the data (*p* > 0.05) before the statistical analysis. Data were statistically analyzed by one-way ANOVA using IBM SPSS software version 21 (SPSS Inc., Chicago, IL, USA), following a completely randomized design. Significant variations between the treatments were evaluated by Tukey’s test (*p* < 0.05). The experimental unit was the replicate for performance measurements and laying hen for other parameters. Additionally, the incremental levels of dietary DOP were tested by orthogonal polynomial contrasts (linear (L), quadratic (Q), and cubic (C) effects), and the *p*-value was recorded. All measured values are shown as mean ± SEM. The Pearson correlation test was applied to identify significant correlations between blood and yolk parameters (*p* < 0.05).

## 3. Results

### 3.1. Laying Performance

Data on the effect of dietary DOP on laying performance are presented in Table 3. There was a non-variation (*p* > 0.05) in the initial body weight among laying hens. The final body weight and weight gain of laying hens fed diets containing DOP at a level of 7 and 10% (DOP_7%_ and DOP_10%_) were greater (*p* < 0.01) than those fed the control and DOP_5%_ diets; this effect was supported by a linear response (*p* < 0.001). The egg production percent during the trial period (from week 25 to week 33 of age) was improved (*p* < 0.001; L, *p* < 0.001; C, *p* = 0.001) in the DOP_7%_ and DOP_10%_ groups compared to those of the control and DOP_5%_ groups. Egg weight and egg mass were increased (*p* < 0.001) in the DOP_7%_ and DOP_10%_ groups compared to the other treatment groups; this influence was indicated by linear (*p* < 0.001), quadratic (*p* < 0.05), and cubic (*p* < 0.001) responses. The incremental levels of DOP in the diets of laying hens enhanced FI and augmented the FCR of hens compared to the control; this response was supported by linear, quadratic, and cubic effects (*p* < 0.001). 

### 3.2. Egg Quality Traits

The effect of the dietary inclusion of DOP on the egg quality traits at the end of the trial is shown in Table 4. There were non-significant differences *(p* > 0.05) in the egg shape index, yolk index, or Haugh unit between the experimental treatments. Eggs obtained from the DOP_7%_ and DOP_10%_ hens had a heavier shell weight% (*p* < 0.01; L, *p* < 0.01) and greater yolk color score (*p* < 0.01; L, *p* < 0.001; C, *p* < 0.05) than those of the control and DOP_5%_ groups. Moreover, eggs from the 10% DOP laying hens had a greater yolk weight% (*p* < 0.01) and shell thickness (*p* < 0.05), but smaller albumen% (*p* < 0.01) compared to other treatment groups. Eggs obtained from the DOP_7%_ hens had a greater albumen index (*p* < 0.01) than the other groups; this effect was supported by a quadratic response (*p* < 0.001).

### 3.3. Reproductive Tract Morphology

At the end of the trial (week 33 of age), the relative weights of the ovary and oviduct of hens receiving diets containing 7% and 10% DOP were heavier (*p* < 0.01) than those of the control and DOP_5%_ hens; this was supported by linear (*p* < 0.01) and quadratic (*p* < 0.05) effects (Table 5). The DOP groups exhibited a greater relative weight of the uterus and follicular weight (*p* < 0.01) compared to those fed the control diet; this was indicated by a linear response (*p* < 0.01) due to the dietary inclusion of DOP. The number of large yellow follicles was increased (*p* < 0.001; L, *p* < 0.001) in the hens-fed diets containing 7% and 10% DOP compared to the control and the DOP_5%_ groups (Table 5). The DOP_10%_ hens had a greater number of small yellow follicles (*p* < 0.01) than the other treatments; this impact was indicated by a linear response (*p* < 0.01) due to the addition of DOP in the diets of laying hens. On the contrary, there were non-significant variations *(p* > 0.05) in the follicular diameter, ovarian stroma weight, or the number of white follicles among the treatment groups (Table 5).

### 3.4. Serum Biochemical Constituents

Data on the effects of DOP on serum biochemical constituents are presented in Table 6. There was an increase in the serum concentrations of total protein (*p* < 0.001; L, *p* < 0.001; quadratic, *p* < 0.001) and albumin (*p* < 0.001; L, *p* < 0.001; quadratic, *p* < 0.001) in the DOP_7%_ and DOP_10%_ laying hens, whereas the serum globulin level was increased in the DOP_10%_ group only (*p* < 0.01; C, *p* < 0.01) compared to the other treatment groups. Dietary DOP inclusion had obvious linear and quadratic effects concerning the levels of serum total lipids (L, *p* < 0.001; Q, *p* < 0.05) and total cholesterol contents (L, *p* < 0.001; Q, *p* < 0.01), with reduced levels noticed for laying hens that consumed the DOP_7%_ and DOP_10%_ diets compared to those fed the control diet (*p* < 0.001). Moreover, linear (*p* < 0.001), quadratic (*p* < 0.01), and cubic (*p* < 0.001) responses were noticeable for the serum triglycerides and LDL cholesterol concentrations, whereby lower levels were recorded in the DOP-fed hens compared with those fed the control diet (*p* < 0.001). On the other hand, the HDL cholesterol level was increased (*p* < 0.001; L, *p* < 0.001; Q, *p* < 0.01) in all DOP-fed groups compared with the control ones. Serum obtained from the DOP-supplemented groups had a lower AST level (*p* < 0.01) than the other groups, while there was a non-significant difference *(p* > 0.05) in the serum ALT level among the treatment groups. The serum MDA values were linearly (*p* < 0.001), quadratically (*p* < 0.05), and cubically (*p* < 0.01) decreased (control vs. DOP, *p* < 0.001) in the DOP_7%_- and DOP_10%_-fed groups compared to the control group. On the other hand, the serum concentration of GPx was improved (*p* < 0.001; L, *p* < 0.001; C, *p* < 0.01) for laying hens fed the DOP_7%_ and DOP_10%_ diets compared to those fed the control diet.

### 3.5. Egg Yolk Fatty Acid Profile

The dietary DOP inclusion linearly (*p* < 0.001) and quadratically (*p* < 0.05) improved the egg yolk concentrations of PUFA, n-3 PUFA, and n-6 PUFA, whereas the content of SFA was reduced (*p* < 0.001; L, *p* < 0.001; Table 7). The relative content of the egg yolk n-3 PUFA was improved by about 33.16%, 53.1%, and 88.27% in the DOP_5%_, DOP_7%_, and DOP_10%_ groups, respectively, compared to the control. On the other hand, the egg yolk MUFA was not different *(p* > 0.05) among the experimental groups. There was a decrease (*p* < 0.001) in the n-6 to n-3 PUFA ratio in all the DOP-fed groups compared to the control group; this effect was supported by linear (*p* < 0.001), quadratic (*p* < 0.001), and cubic (*p* < 0.01) responses due to the incremental levels of DOP in the diets of laying hens (Table 5). The ratios of MUFA:SFA and PUFA:SFA in the egg yolk of laying hens fed DOP diets were greater (*p* < 0.001) than those fed the control diet; this response was supported by a linear response for MUFA:PUFA (*p* < 0.001) and linear and quadratic responses for PUFA:SFA (*p* < 0.01).

### 3.6. Egg Yolk Triglyceride, Cholesterol, and Oxidative Stability

**Figure 1:**
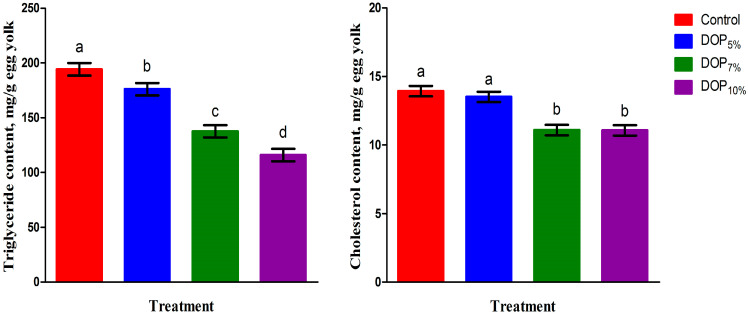
Cholesterol and triglyceride contents in egg yolk of laying hens fed different levels of dried orange pulp (DOP). ^abcd^ Means with dissimilar superscripts within each parameter differ at *p* < 0.05; SEM = Standard error of the mean. The experimental diets were a control diet (no DOP) and experimental diets containing 50, 70, or 100 g DOP/kg feed (DOP_5%_, DOP_7%_, and DOP_10%_, respectively).

**Figure 2:**
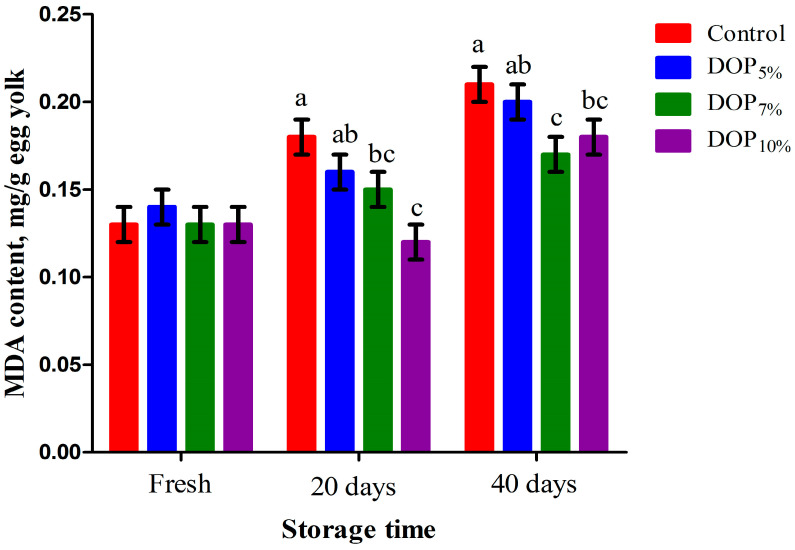
Malondialdehyde (MDA) concentration in fresh and stored eggs of laying hens fed different levels of dried orange pulp (DOP). ^abc^ Means with dissimilar superscripts within each parameter differ at *p* < 0.05; SEM = Standard error of the mean. The experimental diets were a control diet (no DOP) and experimental diets containing 50, 70, or 100 g DOP/kg feed (DOP_5%_, DOP_7%_, and DOP_10%_, respectively).

**Figure 3:**
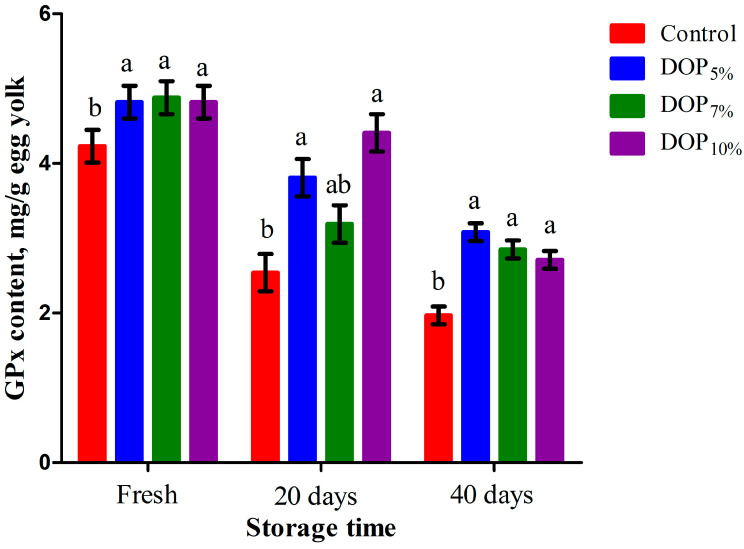
Glutathione peroxidase (GPx) concentration in fresh and stored eggs of laying hens fed different levels of dried orange pulp (DOP). ^ab^ Means with dissimilar superscripts within each parameter differ at *p* < 0.05; SEM = Standard error of the mean. The experimental diets were a control diet (no DOP) and experimental diets containing 50, 70, or 100 g/kg dried orange pulp (DOP_5%_, DOP_7%_, and DOP_10%_, respectively).

Figure 1 presents triglyceride and cholesterol concentrations in the egg yolk of laying hens fed the control and DOP diets. The triglyceride content was linearly reduced (*p* < 0.001) in the egg yolk of all DOP-fed groups compared to the control ones (*p* < 0.001). The concentration of egg yolk cholesterol was lower (*p* < 0.001) for laying hens fed the DOP_7%_ and DOP_10%_ diets than the control ones; this was implied by both linear and quadratic effects (*p* < 0.01). The egg yolk MDA and GPx contents of laying hens fed the control and DOP diets during the storage period are presented in Figure 2 and Figure 3, respectively. The MDA contents in the egg yolk after storage for 20 and 40 days were linearly and quadratically reduced (*p* < 0.01) due to the DOP inclusion in the diets of laying hens. On the other hand, DOP inclusion in the diets of laying hens linearly, quadratically, and cubically increased (*p* < 0.01) the GPx content after storage for 20 and 40 days in the egg yolk of such hens when compared to the control.

### 3.7. Pearson Correlation between Blood and Egg Yolk Parameters

Table 8 shows Pearson correlations between both serum and yolk lipid profiles and antioxidant-related indicators. Serum cholesterol was negatively correlated with serum GPx (r = −0.962, *p* < 0.001), but positively correlated with serum triglyceride (r = 0.963, *p* < 0.001) and MDA (r = 0.918, *p* < 0.001) levels. Additionally, egg yolk cholesterol had positive correlations with serum cholesterol (r = 0.914, *p* < 0.001), triglyceride (r = 0.912, *p* < 0.001), and MDA (r = 0.883, *p* < 0.001) levels, but it negatively correlated with both serum (r = −0.965, *p* < 0.001) and egg yolk (r = −0.617, *p* < 0.05) GPx. Furthermore, egg yolk GPx had a negative relationship with serum triglyceride content (r = −0.603, *p* < 0.05) and a positive relationship with serum GPx concentration (r = 0.640, *p* < 0.05). Regarding the FA profile in egg yolk, yolk PUFA positively correlated with yolk n-6 FA (r = 0.986, *p* < 0.001) and n-3 FA (r = 0.874, *p* < 0.001) contents. Yolk SFA negatively correlated with yolk PUFA (r = −0.844, *p* < 0.01), n-6 FA (r = −0.765, *p* < 0.01), and n-3 FA (r = −0.927, *p* < 0.001) contents. A positive relationship (r = 0.779, p = 0.003) was observed between n-6 and n-3 FA in egg yolk. Yolk n-3 FA had a positive correlation with both serum (r = 0.888, *p* < 0.001) and yolk (r = 0.645, *p* < 0.05) GPx contents, but it negatively correlated with serum MDA concentration (r = −0.730, *p* = 0.007).

## 4. Discussion

To our knowledge, this is the first report on the effects of graded dietary inclusion levels of DOP on the laying rate, egg quality, reproduction morphology, oxidative stability, and egg yolk FA profile in laying hens during the early phase of egg production. The dietary inclusion of DOP at a level of 7% and 10% improved feed intake, egg production, egg mass, and FCR compared to the control and DOP_5%_ groups. Our results agreed with the findings of Yang and Chung [36] and Nazok et al. [23]. Karunajeewa [37] observed that egg production was not affected by the inclusion of 5% citrus pulp in laying hens’ diets. Florou-Paneri et al. [38] found that the inclusion of DCP at a level of 6% in the laying quails’ diet had no negative impact on egg production. Nazok et al. [23] reported the inclusion of DCP at 12–16% in the diets of laying hens caused a significant reduction in the egg production, egg mass, FCR, and final body weight of laying hens. They admitted this decrease in the laying rate was due to the increase in dietary CF content. Ojabo et al. [39] recorded adverse effects on FI, FCR, and body weight in the pullets-fed diet containing 10% orange peel pulp. Goliomytis et al. [24] reported a reduction in FI, laying rate, and FCR in laying hens fed 9% DOP, and they suggested that the reduced values of FI may be attributed to the low palatability of DOP, rather than to the dietary CF content. Based on previous research, the current findings can indicate that dietary inclusion of DOP up to 10% did not adversely affect the palatability of the diets. However, in the above-mentioned studies, they did not report the citrus species from which citrus pulp was obtained and whether seeds were involved in it. The involvement of seeds in citrus pulp or the various citrus species may be accountable for the difference in the level of anti-nutritional constituents, such as tannins and pectin, which may consequently clarify inconsistencies among studies.

In the current study, the CF contents were 2.93%, 3.45%, 3.49%, and 3.60% in the control, DOP_5%_, DOP_7%_, and DOP_10%_ diets, respectively. The addition of moderate levels of CF in the diet of laying hens improves nutrient digestibility and laying performance [2]. Overall, the present results give further provision for the use of DOP at up to 10% in the diet of laying hens (35 g CF/kg of diet) without negatively affecting their laying performance during the early phase of production. Chickens generally adapt to CF-rich diets by enlarging their digestive tract volume [40,41], and consequently augmenting their FI and growth performance [2,41]. This is perhaps one of the causes underlying the better laying rate of hens fed diets having DOP up to 100 g/kg, since this improvement was associated with an increase in FI. Inadequate nutrient intake did not permit laying hens to prompt their genetic potential for egg production [24]. In support of this, the current study verified the amelioratory activity of DOP on ovarian follicles of laying hens, particularly the number and weight of follicles. Nevertheless, the DOP mechanism of action is not yet determined; we proposed that DOP may augment follicular growth and development, possibly because of its valuable dietary constituents, including flavonoid and phenolic compounds. To our knowledge, there is currently no more research reported about the effects of DOP on the morphology of the reproductive tract to compare with the results recorded herein. 

Dietary inclusion of DOP linearly increased the egg weight, shell weight%, shell thickness, and yolk color score, particularly in the eggs obtained from the DOP_7%_ and DOP_10%_ groups. Nazok et al. [23] reported that the utilization of DCP at up to 16% did not affect the eggshell thickness, the eggshell index, or the yolk color score. Moreover, Goliomytis et al. [24] recorded deterioration in egg quality traits, such as egg weight, shell thickness and strength, and yolk color, in eggs obtained from laying hens fed 9% DOP. Our findings partially agreed with Nazok et al. [23], who observed a higher egg weight and egg mass from hens fed 8, 12, and 16% citrus pulp. The reduction in FI observed in the study by Goliomytis et al. [24] not only affected the laying rate and FCR, but also caused some of the egg quality traits to deteriorate. In the current study, the improved FI in the DOP-fed groups, and thus sufficient nutrient supply, led to an increase in egg production, egg mass, egg weight, and egg yolk weight. Our findings agreed with Florou-Paneri et al. [38], who found that the inclusion of DCP at up to 6% in the laying quails’ diet had no adverse effect on egg weight. The fact that these authors did not report any improvement in FI or egg quality traits due to citrus pulp utilization supports the assumption that the enhancement of some egg quality traits in the current trial may be attributed to the better FI of the DOP-fed groups. In agreement with the present study, Oyewole et al. [42] reported a heavier egg weight in laying hens fed 10% to 40% dried sweet orange peel. Furthermore, Ahmed et al. [43] recorded improved FCR, egg mass, and egg weight with the addition of 5% and 10% dried orange peel in the diet of laying hens.

For the egg industry and consumer preferences, yolk color is an essential feature of egg quality. Consumers prefer golden, orange, and pigmented egg yolk [44]. The orange pulp contains a considerable content of carotenoids and xanthophyll [45], which may be responsible for better yolk color in the DOP-fed groups. However, we did not analyze the total carotenoid content of DOP in this study to confirm this assumption. Angalet et al. [46] found additional orange yolk color with an increase in the dietary levels of citrus sludge from 2.5% to 20% in hens. Chowdhury et al. [47] observed higher egg yolk color in hens fed 4% orange skin. The improvement in the eggshell% and thickness in the current study, particularly in DOP_10%_, may be due to the adequate dietary Ca intake, which is beneficial for eggshell formation as a result of the increased FI of the DOP_10%_ laying hens. The study by Goliomytis et al. [24] observed deterioration in the shell thickness and shell strength due to DOP inclusion at a level of 90 g/kg of the diet. These authors attributed this effect to inadequate dietary Ca intake because of reduced FI.

One of the main aims of the current trial was to determine the egg yolk oxidative stability of laying hens fed DOP. Our findings showed that the dietary inclusion of DOP for 8 weeks was efficient in enhancing the antioxidant activities (GPx) and lowering the MDA contents of both fresh and stored eggs for 40 days, and subsequently extended its shelf life. It was assumed that the exerted antioxidant activities of DOP on the egg yolk can be attributed to its hesperidin and naringin contents [24]. Hesperidin and naringin presented in DOP can diminish radical chain reactions, in the egg yolk lipid portion, by donation of hydrogen atoms to free radicals simulating the antioxidant properties of α-tocopherol [24,48]. The positive effects of dietary hesperidin and naringin [24,49] or DOP [24] on egg yolk antioxidant activities, oxidative stability, and shelf life have been recorded [24,49]. In line with this, the current findings showed that the egg yolk cholesterol level was positively correlated with the serum MDA level, but negatively correlated with both the serum and yolk GPx levels. Dietary orange peel and grapefruit peel were reported to effectively decrease the oxidation process in the meat of broiler chickens following storage [20]. Recently, Zoidis et al. [22] observed that dietary supplementation of DOP at 50 g/kg, along with organic Se, improved meat oxidative stability and nutritive value. Flavonoids and vitamin C present in DOP have beneficial antioxidant properties [13,14] and antimicrobial [15,16,17] and chelating actions [13,14]. We can declare that orange pulp waste management can help to diminish egg MDA levels, augment the antioxidant status of eggs, and subsequently extend egg shelf life. At the same time, more research is necessary to measure carotenoid contents in the egg yolks from laying hens fed diets containing DOP to support the assumption of the present study.

The inclusion of DOP in the diets of laying hens induced positive effects on blood cholesterol, triglyceride, LDL, and HDL contents, as well as egg yolk cholesterol and triglyceride levels. These findings are consistent with Abbasi et al. [19], who observed that the utilization of dried sweet orange pulp in the diets of broiler chickens at up to 2% reduced blood triglycerides, total cholesterol, and LDL cholesterol. Nazok et al. [23] found that the use of dietary DCP in the diets of laying hens at more than 16% increased blood serum HDL, while it decreased blood cholesterol, LDL, and triglyceride contents. The decrease in cholesterol levels may be attributed to the diminishing of HMG-CoA reductase activity, which is the main enzyme in cholesterol biosynthesis [19,50]. The inhibition of this enzyme enhances LDL receptors and improves blood HDL levels [50,51]. Likewise, some suggestions proposed that hesperidin works as a cholesterol-lowering factor by reducing the hepatic activity of HMG-CoA reductase [50]. Moreover, the elevated level of soluble fiber may induce an adverse influence on lipid digestion by stimulating the synthesis of bile salts and fiber complex [52]. This complex can lessen cholesterol levels in both the serum and egg yolk of hens [23]. Additionally, citrus fruits were reported to contain an abundant quantity of pectin, which appears both in the edible and inedible portions of fruit [53]. Numerous mechanisms have been intended for the cholesterol-lowering effect of pectin. Pectin was reported to reduce pancreatic enzyme activity, which in sequence could increase fecal lipid excretion [54]. Nevertheless, additional evidence is required to fully clarify the potential mechanism of the cholesterol-lowering effect of DOP.

Blood AST is a useful marker of hepatic function and health. An elevated blood AST level is a reflexive reaction of an organism to hepatic inflammation and injury [55]. It has been observed that the utilization of citrus maxima peel in the diet of mice with CC14-induced hepatic damage significantly reduced both serum and hepatic AST levels [56]. The serum level of AST was decreased in the DOP-supplemented groups compared to the control, while there was no change in the serum ALT concentration. These results suggested that the hepatic function was not adversely affected by the dietary inclusion of DOP at up to 10% in laying hens. The current findings agreed with Nazok et al. [23], Abbasi et al. [19], and Lu et al. [57]. Blood proteins are components of the immune response, in which antibodies are composed. In this study, serum total proteins, albumin, and globulin were significantly increased in the DOP-fed hens, which implied a better health status of laying hens in the DOP groups. Higher plasma albumin content and the albumin to globulin ratio were recorded in the dried citrus pulp-fed rabbits [57,58], revealing the same trend in the current trial.

Dietary modification by DOP inclusion significantly augmented the egg yolk FA profile due to the improvement in the health-promoting n-3 FA and PUFA contents and a reduction in SFA content. An average increase in the egg yolk n-3 PUFA content by 33.16%, 53.1%, and 88.27% was determined among the DOP-fed groups and the control groups. Consequently, the inclusion of laying hens’ diets with DOP not only enhanced the antioxidant properties of the fresh and stored egg yolks, but also boosted their nutritional value through enrichment with beneficial n-3 FA and PUFA. The noticeable increase in the n-3 and n-6 PUFA contents may be attributed to the increased PUFA content of the DOP diets. At the same time, the improved PUFA content in the egg yolk of laying hens fed with DOP may be attributed to the protective effect of the DOP phenolic and flavonoids on PUFA from oxidation. This was supported by the observed positive relationship among yolk PUFA, n-6 FA, and n-3 FA contents, as well as a negative correlation between SFA and PUFA (also n-6 and n-3 FA). Furthermore, yolk n-3 FA correlated positively with both the serum and yolk GPx contents, but negatively with the serum MDA concentration. An increase in PUFA content and a decrease in both MUFA and SFA in meat has been recorded in ostriches fed with DCP at 20% of the diet [59]. Moreover, Zoidis et al. [22] observed an increase in the n-3 FA and MUFA content, whereas the SFA content slightly decreased in the breast meat of broiler chickens fed with DOP at 50 g/kg of the feed. Unfortunately, no data exist on the effect of dietary inclusion of DOP in layers on the FA composition of the egg yolk to compare with the findings reported herein.

## 5. Conclusions

According to the results obtained in the current study, dietary DOP inclusion at up to 100 g/kg of the feed improved the antioxidant capacity (augmented GPx and diminished MDA) of the fresh and stored eggs, with the enhancement being more obvious in long-term storage of up to 40 days. Further, dietary modification by DOP inclusion augmented the egg’s nutritional value via the enrichment with PUFA—more prominently, the health-promoting n-3 PUFA—and the reduction in egg yolk SFA, cholesterol, and triglyceride contents. Moreover, the relative weights of the ovary, oviduct, uterus, and follicle, as well as the number of large yellow follicles, were increased in the hens-fed diets containing 7% and 10% DOP. At the same time, these favorable effects found in the current trial were associated with beneficial outcomes on the laying rate, egg quality traits, and bird’s health status. These findings indicated that the addition of DOP to laying hens’ diets at up to 100 g/kg of feed can improve the laying performance, health status, antioxidant capacity, egg nutritive value, and egg shelf life of laying hens, with profits for both the farmer and the consumer. Further studies are needed to determine the DOP mechanism of action on follicular growth and development, as well as its cholesterol-lowering effect.

## Figures and Tables

**Table 1:** Nutrient composition of dried orange pulp (DOP).

Items ^1^	DOP
ME, kcal/kg	2724.67
DM, %	91.9
CP, %	6.9
Ca, %	2.23
AP, %	0.117
CF, %	12.5
NDF, %	25.4
ADF, %	21.8
EE, %	3.37
Total phenolic, mg GAE/100 g	2300
Total flavonoid, mg CE/100 g	463

^1.^ ME = metabolizable energy, DM = dry matter, CP = crude protein, Ca = calcium, AP = available phosphorus, CF = crude fiber, NDF = neutral detergent fiber, ADF = acid detergent fiber, EE = ether extract.

**Table 2:** Ingredients, proximate composition, and FA profile of the experimental diets.

Items	Control	DOP_5%_	DOP_7%_	DOP_10%_
Ingredients, %				
Yellow corn	54.57	49.37	49.57	49.61
Soybean meal, 44% CP	24.90	25.00	25.42	25.80
Wheat bran	8.50	8.50	6.50	3.91
DOP	0.00	5.00	7.00	10.00
Vegetable oil	2.10	2.50	2.00	1.30
Dicalcium phosphate	1.73	1.73	1.71	1.78
Limestone	7.50	7.20	7.10	6.90
Premix ^1^	0.40	0.40	0.40	0.40
Common salt	0.30	0.30	0.30	0.30
Total	100	100	100	100
Chemical composition				
ME, MJ/kg	11.39	11.39	11.39	11.39
DM, %	91.30	91.40	91.10	91.60
CP, %	17.15	17.13	17.16	17.14
Ca, %	3.33	3.33	3.33	3.33
AP, %	0.45	0.45	0.44	0.44
CF, %	2.93	3.45	3.49	3.60
Methionine, %	0.49	0.49	0.50	0.50
Lysine, %	0.88	0.89	0.89	0.90
Fatty acid profile, %				
SFA	22.60	20.15	19.86	19.31
MUFA	27.47	27.05	27.30	27.65
PUFA	49.93	52.81	52.84	53.04
n-6 FA	46.01	48.76	48.39	48.00
n-3 FA	3.92	4.05	4.44	5.04
PUFA:SFA	2.21	2.62	2.66	2.75
MUFA:SFA	1.22	1.34	1.37	1.43
n-6:n-3	11.73	12.05	10.89	9.53

^1^ Provided per kg of the diet: 13,000 IU vit A, 3500 IU vit D, 45 mg vit E, 3.5 mg vit K, 2 mg vit B_1_, 6 mg vit B_2_, 6.1 mg; 45 mg vit B_3_, 12 mg vit B_5_, 5 mg vit B_6_, 0.08 vit B_7_, 2 mg vit B_9_, 0.02 mg vit B_12_; 0.075 mg, 100 mg Manganese, 600 mg Zinc, 30 mg iron, 10 mg Cu, 1 mg iodine, 0.2 mg selenium, 0.1 mg cobalt. SFA, saturated fatty acid; MUFA, monounsaturated fatty acid; PUFA, polyunsaturated fatty acid; FA, fatty acid.

**Table 3:** Production performance of laying hens fed the experimental diets.

Items	Treatments ^1^				SEM	Significance, *p*-Value			
	Control	DOP_5%_	DOP_7%_	DOP_10%_		ANOVA	L	Q	C
Body weight (BW), g									
Initial BW	1335.66	1338.73	1340.74	1336.15	11.97	0.97	-	-	-
Final BW	1710.53 ^b^	1724.31 ^ab^	1755.41 ^a^	1765.64 ^a^	16.97	0.005	˂0.001	0.88	0.48
BW gain	374.88 ^c^	385.58 ^bc^	414.67 ^ab^	429.49 ^a^	14.21	0.001	˂0.001	0.83	0.47
Egg production, %									
W25 to W29	88.30 ^b^	88.40 ^b^	91.99 ^a^	91.31 ^a^	1.08	0.001	˂0.001	0.61	0.03
W29 to W33	91.04 ^b^	92.35 ^b^	97.71 ^a^	97.07 ^a^	0.93	˂0.001	˂0.001	0.14	0.001
W25 to W33	89.67 ^b^	90.38 ^b^	94.85 ^a^	94.19 ^a^	0.82	˂0.001	˂0.001	0.24	0.001
Egg weight, g									
W25 to W29	50.26 ^c^	50.67 ^bc^	51.56 ^a^	50.99 ^ab^	0.24	˂0.001	˂0.001	0.006	0.013
W29 to W33	52.31 ^b^	52.53 ^b^	53.63 ^a^	52.80 ^b^	0.23	˂0.001	0.001	0.002	˂0.001
W25 to W33	51.29 ^c^	51.60 ^bc^	52.59 ^a^	51.89 ^b^	0.19	˂0.001	˂0.001	˂0.001	˂0.001
Egg mass, g									
W25 to W29	44.38 ^b^	44.78 ^b^	47.44 ^a^	46.57 ^a^	0.62	˂0.001	˂0.001	0.15	0.004
W29 to W33	47.63 ^b^	48.50 ^b^	52.41 ^a^	51.25 ^a^	0.55	˂0.001	˂0.001	0.01	˂0.001
W25 to W33	45.99 ^b^	46.63 ^b^	49.89 ^a^	48.88 ^a^	0.47	˂0.001	˂0.001	0.01	˂0.001
Feed intake, g/d									
W25 to W29	107.14 ^b^	109.16 ^a^	109.39 ^a^	108.62 ^a^	0.32	˂0.001	˂0.001	˂0.001	0.44
W29 to W33	114.27 ^c^	118.16 ^b^	117.91 ^b^	119.12 ^a^	0.34	˂0.001	˂0.001	˂0.001	˂0.001
W25 to W33	110.70 ^b^	113.66 ^a^	113.65 ^a^	113.87 ^a^	0.27	˂0.001	˂0.001	˂0.001	˂0.001
FCR, g feed/g egg mass									
W25 to W29	2.42 ^a^	2.44 ^a^	2.31 ^b^	2.34 ^b^	0.032	˂0.001	˂0.001	0.94	0.003
W29 to W33	2.40 ^a^	2.44 ^a^	2.25 ^c^	2.33 ^b^	0.028	˂0.001	˂0.001	0.34	˂0.001
W25 to W33	2.41 ^a^	2.44 ^a^	2.28 ^b^	2.33 ^b^	0.024	˂0.001	˂0.001	0.51	˂0.001

^abc^ Means with dissimilar superscripts within each parameter differ at *p* < 0.05; SEM = Standard error of the mean. ^1^ The experimental diets were a control diet (no DOP) and experimental diets containing 50, 70, or 100 g DOP/kg feed (DOP_5%_, DOP_7%_, and DOP_10%_, respectively). FCR, feed conversion ratio.

**Table 4:** Egg quality traits of laying hens fed the experimental diets.

Items	Treatments ^1^				SEM	Significance, *p*-Value			
	Control	DOP_5%_	DOP_7%_	DOP_10%_		ANOVA	L	Q	C
Egg shape index	81.36	82.32	83.05	80.97	0.82	0.06	0.86	0.01	0.32
Shell weight, %	14.23 ^b^	14.76 ^ab^	15.09 ^a^	15.09 ^a^	0.25	0.005	0.001	0.15	0.85
Albumen weight, %	62.35 ^a^	62.30 ^a^	62.87 ^a^	60.09 ^b^	0.81	0.009	0.02	0.02	0.13
Albumen index	11.21 ^b^	11.61 ^ab^	11.89 ^a^	11.06 ^b^	0.21	0.002	0.79	0.000	0.14
Yolk weight, %	23.42 ^ab^	22.94 ^b^	22.04 ^b^	24.82 ^a^	0.74	0.007	0.17	0.004	0.09
Yolk index	69.53	69.99	70.32	71.50	1.05	0.30	0.07	0.63	0.77
Yolk color score	5.38 ^b^	5.54 ^b^	6.86 ^a^	7.33 ^a^	0.25	˂0.001	˂0.001	0.38	0.02
Haugh unit	81.04	81.39	81.36	80.78	0.25	0.06	0.31	0.01	0.82
Shell thickness, mm	0.371 ^b^	0.384 ^ab^	0.380 ^ab^	0.383 ^a^	0.004	0.03	0.05	0.14	0.08

^ab^ Means with dissimilar superscripts within each parameter differ at *p* < 0.05; SEM = Standard error of the mean. ^1^ The experimental diets were a control diet (no DOP) and experimental diets containing 50, 70, or 100 g DOP/kg feed (DOP_5%_, DOP_7%_, and DOP_10%_, respectively).

**Table 5:** Reproductive tract morphology of laying hens fed the experimental diets.

Items	Treatments ^1^				SEM	Significance, *p*-Value			
	Control	DOP5%	DOP7%	DOP10%		ANOVA	L	Q	C
Ovary weight, g	38.83 ^c^	40.78 ^bc^	43.86 ^a^	42.75 ^ab^	0.83	˂0.001	˂0.001	0.02	0.07
Relative ovary weight, %	2.22 ^c^	2.28 ^bc^	2.44 ^a^	2.34 ^ab^	0.02	˂0.001	˂0.001	˂0.001	˂0.001
Large yellow follicles, N	5.33 ^c^	6.00 ^bc^	6.33 ^b^	7.33 ^a^	0.29	˂0.001	˂0.001	0.43	0.29
Small yellow follicles, N	8.67 ^b^	9.00 ^b^	9.33 ^ab^	10.33 ^a^	0.41	0.009	0.001	0.27	0.61
Large white follicles, N	10.33	12.67	9.33	12.33	1.42	0.11	0.56	0.75	0.02
Oviduct weight, g	57.34 ^c^	63.25 ^b^	68.27 ^a^	66.19 ^ab^	1.42	˂0.001	˂0.001	0.002	0.19
Relative oviduct weight, %	3.28 ^b^	3.54 ^ab^	3.79 ^a^	3.63 ^a^	0.10	0.002	0.001	0.009	0.22
Follicle weight, g	8.73 ^b^	11.04 ^a^	11.29 ^a^	11.43 ^a^	0.56	0.001	0.001	0.018	0.29
Follicle diameter, mm	37.08	38.15	35.76	36.76	1.39	0.43	0.46	0.97	0.15
Uterus weight, g	6.83 ^b^	11.46 ^a^	10.30 ^a^	12.66 ^a^	1.14	0.002	0.001	0.18	0.02
Relative uterus weight, %	0.39 ^b^	0.64 ^a^	0.57 ^ab^	0.69 ^a^	0.06	0.003	0.001	0.18	0.03
Ovarian stroma weight, g	4.52	4.75	4.92	4.63	0.14	0.06	0.28	0.02	0.35
Relative ovarian stroma, %	0.26	0.27	0.27	0.25	0.01	0.14	0.77	0.04	0.30

^abc^ Means with dissimilar superscripts within each parameter differ at *p* < 0.05; SEM = Standard error of the mean. ^1^ The experimental diets were a control diet (no DOP) and experimental diets containing 50, 70, or 100 g DOP/kg feed (DOP_5%_, DOP_7%_, and DOP_10%_, respectively).

**Table 6:** Serum biochemical constituents of laying hens fed the experimental diets.

Items	Treatments ^1^				SEM	Significance, *p*-Value			
	Control	DOP5%	DOP7%	DOP10%		ANOVA	L	Q	C
Protein metabolites									
Total protein, g/dL	4.20 ^d^	4.65 ^c^	5.00 ^b^	5.31 ^a^	0.093	˂0.001	˂0.001	0.29	0.84
Albumin, g/dL	2.07 ^c^	2.21 ^b^	2.91 ^a^	2.83 ^a^	0.04	˂0.001	˂0.001	0.005	˂0.001
Globulin, g/dL	2.13 ^bc^	2.44 ^ab^	2.10 ^c^	2.48 ^a^	0.099	0.008	0.06	0.63	0.002
A/G ratio	0.97 ^bc^	0.91 ^c^	1.39 ^a^	1.14 ^b^	0.063	˂0.001	0.001	0.08	˂0.001
Lipid metabolites									
Total lipids, mg/dL	392.71 ^a^	377.81 ^ab^	362.64 ^b^	368.04 ^b^	5.02	0.002	0.000	0.02	0.23
Total cholesterol, mg/dL	196.97 ^a^	189.47 ^a^	154.48 ^b^	151.49 ^b^	3.854	˂0.001	˂0.001	0.43	0.001
Triglycerides, mg/dL	90.51 ^a^	82.81 ^b^	63.82 ^c^	66.38 ^c^	1.532	˂0.001	˂0.001	0.001	˂0.001
LDL, mg/dL	64.22 ^a^	60.23 ^b^	40.33 ^c^	42.16 ^c^	0.867	˂0.001	˂0.001	0.001	˂0.001
HDL, mg/dL	35.49 ^c^	43.82 ^b^	49.72 ^a^	51.25 ^a^	1.036	˂0.001	˂0.001	0.002	0.57
Liver function									
ALT, U/L	26.55	25.71	25.89	26.18	0.502	0.41	0.58	0.15	0.58
AST, U/L	74.97 ^a^	72.05 ^b^	71.07 ^b^	72.23 ^b^	0.814	0.007	0.007	0.008	0.94
Antioxidant status									
GPx, U/mL	3.90 ^b^	4.12 ^b^	4.83 ^a^	4.83 ^a^	0.075	˂0.001	˂0.001	0.07	0.001
MDA, nmol/mL	38.34 ^a^	37.57 ^a^	32.73 ^b^	34.29 ^b^	0.658	˂0.001	˂0.001	0.04	0.001

^abcd^ Means with dissimilar superscripts within each parameter differ at *p* < 0.05; SEM = Standard error of the mean. ^1^ The experimental diets were a control diet (no DOP) and experimental diets containing 50, 70, or 100 g DOP/kg feed (DOP_5%_, DOP_7%_, and DOP_10%,_ respectively). LDL, low-density lipoprotein; HDL, high-density lipoprotein; ALT, alanine aminotransferase; AST, aspartate aminotransferase; GPx, glutathione peroxidase; MDA, malondialdehyde.

**Table 7:** Egg yolk fatty acid profile (% of total FAs) of laying hens fed the experimental diets.

Items	Treatments ^1^				SEM	Significance, *p*-Value			
	Control	DOP_5%_	DOP_7%_	DOP_10%_		ANOVA	L	Q	C
SFA	37.88 ^a^	36.07 ^b^	33.20 ^c^	30.75 ^d^	0.41	˂0.001	˂0.001	0.30	0.29
MUFA	38.28	40.64	40.56	40.26	1.12	0.19	0.14	0.13	0.55
PUFA	23.85 ^bc^	23.30 ^c^	26.25 ^b^	29.00 ^a^	0.78	˂0.001	˂0.001	0.02	0.17
n-6 FA	21.90 ^bc^	20.69 ^c^	23.25 ^ab^	25.32 ^a^	0.69	0.001	0.000	0.01	0.08
n-3 FA	1.96 ^c^	2.61 ^b^	3.00 ^b^	3.69 ^a^	0.15	˂0.001	˂0.001	0.87	0.26
n6:n3	11.20 ^a^	7.93 ^b^	7.76 ^b^	6.87 ^b^	0.22	˂0.001	˂0.001	˂0.001	0.003
MUFA:SFA	1.01 ^c^	1.13 ^bc^	1.22 ^ab^	1.31 ^a^	0.05	˂0.001	˂0.001	0.77	0.95
PUFA:SFA	0.63 ^c^	0.65 ^c^	0.79 ^b^	0.94 ^a^	0.02	˂0.001	˂0.001	0.004	0.11

^abcd^ Means with dissimilar superscripts within each parameter differ at *p* < 0.05; SEM = Standard error of the mean. ^1^ The experimental diets were a control diet (no DOP) and experimental diets containing 50, 70, or 100 g/kg dried orange pulp (DOP_5%_, DOP_7%_, and DOP_10%,_ respectively). SFA, saturated fatty acid; MUFA, monounsaturated fatty acid; PUFA, polyunsaturated fatty acid.

**Table 8:** Pearson correlation between serum biochemical constituents and yolk parameters.

		Serum				Yolk							
		CHOL	TAG	GPx	MDA	MDA	GPx	CHOL	TAG	SFA	MUFA	PUFA	n-6 FA
Serum	CHOL	-											
	TAG	0.963 **	-										
	GPx	−0.962 **	−0.957 **	-									
	MDA	0.918 **	0.920 **	−0.950 **	-								
Yolk	MDA	0.128	0.286	−0.294	0.294	-							
	GPx	−0.477	−0.603 *	0.640 *	−0.512	−0.302	-						
	CHOL	0.914 **	0.912 **	−0.965 **	0.883 **	0.389	−0.617 *	-					
	TAG	0.967 **	0.925 **	−0.953 **	0.852 **	0.108	−0.575	0.896 **	-				
	SFA	0.923 **	0.899 **	−0.904 **	0.789 **	0.205	−0.485	0.885 **	0.949 **	-			
	MUFA	−0.300	−0.463	0.331	−0.308	−0.395	0.325	−0.349	−0.274	−0.500	-		
	PUFA	−0.879 **	−0.751 **	0.837 **	−0.720 **	0.008	0.358	−0.805 **	−0.925 **	−0.844 **	−0.043	-	
	n-6 FA	−0.832 **	−0.683 *	0.771 **	−0.674 *	0.026	0.237	−0.735 **	−0.864 **	−0.765 **	−0.166	0.986 **	-
	n-3 FA	−0.868 **	−0.817 **	0.888 **	−0.730 **	−0.044	0.645 *	−0.871 **	−0.944 **	−0.927 **	0.316	0.874 **	0.779 **

CHOL, cholesterol; TAG, triglyceride; GPx, glutathione peroxidase; MDA, malondialdehyde; SFA, saturated fatty acid; MUFA, monounsaturated fatty acid; PUFA, polyunsaturated fatty acid, FA, fatty acid. *, *p* < 0.05; **, *p* < 0.01.

## Data Availability

The data presented in this study are available on request from the corresponding author.
